# Reducing luminance intensity can improve motion perception in noise

**DOI:** 10.1038/srep43140

**Published:** 2017-02-21

**Authors:** Rémy Allard, Angelo Arleo

**Affiliations:** 1 Sorbonne Universités, UPMC Univ Paris 06, INSERM, CNRS, Institut de la Vision, Paris, France

## Abstract

Visual perception generally improves under brighter environments. For instance, motion sensitivity is known to improve with luminance intensity especially at high temporal frequencies. However, the current study counter-intuitively shows that increasing luminance intensity can impair motion sensitivity in noise. Motion sensitivity was measured with and without noise added to a drifting Gabor patch as a function of the temporal frequency and luminance intensity. As expected, motion sensitivity in absence of noise reached a ceiling performance at a relatively low luminance intensity (about 35 td) for low temporal frequencies and improved with luminance intensity up to the highest luminance intensity tested (353 td) for high temporal frequencies. In noise, reducing mean luminance intensity *facilitated* motion sensitivity (up to a factor of about 1.7) for temporal frequencies up to 7.5 Hz and impaired sensitivity at higher temporal frequencies (15 and 30 Hz). We conclude that reducing luminance intensity is effectively equivalent to applying a low-pass filter, which can improve motion sensitivity in noise to low and middle temporal frequencies. This counterintuitive facilitation effect can be explained by two known properties of the visual system: decreasing luminance intensity impairs the visibility of high temporal frequencies (equivalent to a low-pass filter) and motion detectors are broadly tuned.

In a recent study^1^, we found that artificially applying a temporally low-pass filter to the stimulus (which reduces the effective contrast at high temporal frequencies) could improve motion sensitivity in white noise at low and middle temporal frequencies. The visual system is well known to be more temporally low-pass at lower luminance intensities^2^: in photopic conditions, motion sensitivity is independent of luminance intensity at low temporal frequencies (Weber law) and gradually improves with luminance intensity at high temporal frequencies. As a result, reducing luminance intensity should be equivalent to applying a low-pass filter (reduce the effective contrast of high temporal frequencies), which leads to the counter-intuitive prediction that reducing luminance intensity (e.g., by wearing sunglasses) could improve motion sensitivity in high noise at low and middle frequencies. The present study tested and confirmed this counter-intuitive prediction.

Any filter would not affect the signal-to-noise ratio at the signal frequency since it would affect the signal and noise by the same proportion. It is therefore reasonable to expect motion sensitivity in high noise to be independent of luminance intensity. In fact, spatial contrast sensitivity (i.e., static stimuli) in high noise has been found to be independent of luminance intensity^3^,^4^. However, the visual system is more broadly tuned temporally than spatially^5^ so even though luminance intensity affects similarly the spatial and temporal sensitivity functions (band-pass at high luminance intensities and low-pass at low luminance intensities), we could expect different outcomes. If the noise at high temporal frequencies interferes with the processing of a signal at a much lower frequency, reducing the sensitivity to high temporal frequencies (analogous to applying a low-pass filter) by reducing luminance intensity could improve motion sensitivity. The current study therefore evaluated motion sensitivity (i.e., contrast threshold using direction discrimination task) at various temporal frequencies and luminance intensities in absence of noise and in high noise.

## Results

**Figure 1:**
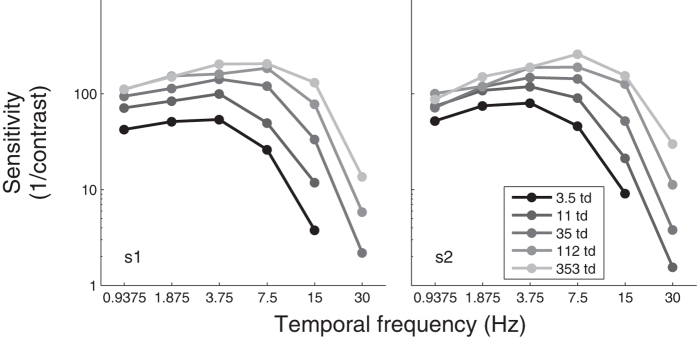
Motion sensitivity (inverse of contrast threshold) as a function of the temporal frequency for different luminance intensities for two observers (left and right graphs).

**Figure 2:**
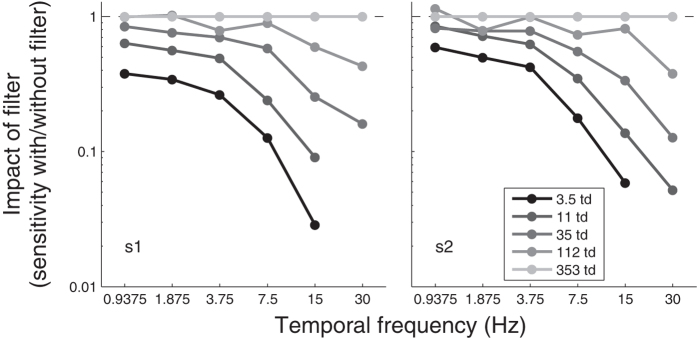
Impact of neutral density filter on motion sensitivity, that is, sensitivity normalized relative to the sensitivity with no filter corresponding to the highest luminance intensity of 353 td. Data re-plotted from Fig. 1.

In absence of noise, the pattern of results for motion sensitivity as a function of luminance intensity and temporal frequency (Fig. 1) was inline with what is typically observed for temporal contrast sensitivity^2^. This was expected given that motion sensitivity and temporal contrast sensitivity are equivalent^6^,^7^. Specifically, at low temporal frequencies, sensitivity was independent of luminance intensity (i.e., Weber law) at high luminance intensities (about >35 td), whereas at high temporal frequencies, sensitivity depended on luminance intensity at all luminance intensities (i.e., up to at least 353 td). In other words, the visual system became more temporally low-pass at lower luminance intensities. To illustrate the greater impact of reducing luminance intensity at high temporal frequencies, Fig. 2 shows the same data normalized relative to the sensitivity at the highest luminance intensity at which no filter were used, which illustrates the impact of different neutral density filters reducing luminance intensity.

**Figure 3:**
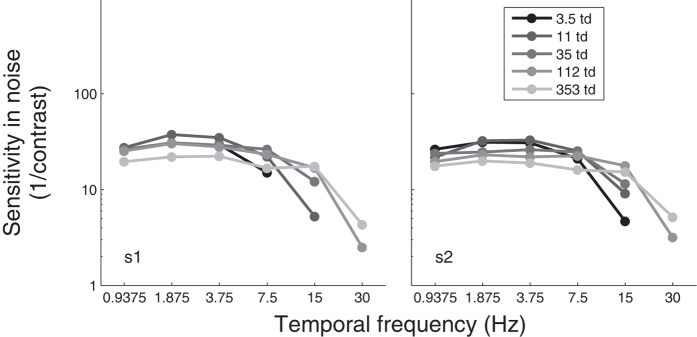
Motion sensitivity (inverse of contrast threshold) in high noise as a function of the temporal frequency for different luminance intensities for two observers (left and right graphs).

**Figure 4:**
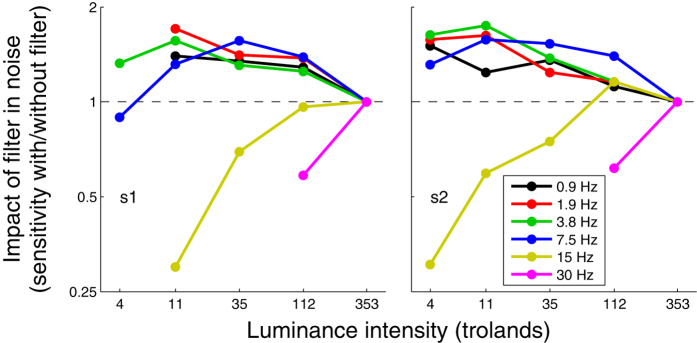
Motion sensitivity (inverse of contrast threshold) in high noise normalized relative to the sensitivity at the highest luminance intensity (353 td) as a function of luminance intensity for different the temporal frequencies for two observers (left and right graphs). The data re-plotted from Fig. 3.

In noise, motion sensitivity varied considerably less with luminance intensity than in absence of noise (curves overlap more in Fig. 3 than in Fig. 1). This is not surprising given that any filter would equally affect the signal and noise at the signal frequency. Nevertheless, motion sensitivity in noise was not independent of luminance intensity as a non-negligible variation remained (the different curves in Fig. 3 do not perfectly overlap). These variations are more obvious in Fig. 4, which plots the sensitivity normalized relative to the sensitivity at the highest luminance intensity (353 td) as a function of luminance intensity. For temporal frequencies up to 7.5 Hz, reducing mean luminance intensity was found to improve motion sensitivity in noise sometimes by considerable proportions as much as a factor of 1.7. Conversely, higher temporal frequencies were impaired by a reduction of luminance intensity.

## Discussion

Motion sensitivity in high noise was found to depend on luminance intensity. Counter-intuitively, decreasing luminance intensity *improved* motion sensitivity in noise at low and middle temporal frequencies. This facilitation effect can be explained by two known properties of the visual system: decreasing luminance intensity impairs the visibility of high temporal frequencies^2^ (equivalent to a low-pass filter) and motion detectors are broadly tuned^5^. Reducing luminance intensity therefore reduced the effective masking strength of noise at high temporal frequencies that is integrated by the broadly tuned motion detectors. Thus, although this motion sensitivity facilitation is counter-intuitive, it is consistent with known properties of the visual system.

Interestingly, the results for motion sensitivity differ from the ones obtained for spatial contrast sensitivity, which was found to be independent of luminance intensity in high noise^3^,^4^. These different outcomes are surprising given that contrast sensitivity varies similarly as a function of spatial and temporal frequency: luminance intensity affects more sensitivity at high than low frequencies resulting in a band-pass sensitivity function at high luminance intensities and low-pass at low luminance intensities. In high noise, the improvement for motion sensitivity and not for spatial contrast sensitivity can be explained by the fact that the visual system is more broadly tuned to temporal than spatial frequencies^5^. As a result, motion sensitivity to low and middle temporal frequencies would be affected by noise at high temporal frequencies, whereas spatial contrast sensitivity to low and middle spatial frequencies would be roughly independent of noise at high spatial frequencies. Thus, reducing luminance intensity removes noise at high temporal and spatial frequencies, but this would only facilitate motion sensitivity, not spatial contrast sensitivity.

At first sight, the fact that neutral density filters (e.g., wearing sunglasses) reducing luminance intensity can improve motion sensitivity in noise seems an interesting practical improvement. However, this advantage is restrained to very specific conditions. The advantage comes from reducing the visibility of irrelevant masking information at high temporal frequencies. Introducing temporal blur (i.e., low-pass temporal filtering) by reducing luminance intensity reduces the visibility of high temporal frequencies, which would be beneficial for perceiving lower temporal frequencies when there are irrelevant masking information (e.g., noise) at high temporal frequencies. An analogous phenomenon has been observed in spatial vision with spatial blur reducing sensitivity to high spatial frequencies. There are particular conditions under which spatial blur can improve visual perception because irrelevant information at high spatial frequencies impairs the perception of information defined by lower spatial frequencies. For instance, blur can improve face recognition from a coarsely sampled picture^8^. In this case, salient edges of the coarsely sampled picture impair recognition of faces defined by lower spatial frequencies, so filtering out these edges by introducing spatial blur can improve visual recognition. In sum, impairing our perception of some visual information would be useful when this information is irrelevant (e.g., noise or artificial edges) and thereby improve our ability to perceive other relevant information (e.g., motion or face recognition at lower frequencies). Practical useful implications of the current finding are therefore restrained to particular conditions.

## Method

### Observers

Two naïve observers who had corrected-to-normal vision participated to the study. Observers worn trial lens and stimuli were viewed monocularly with their dominant eye and the non-dominant eye was closed and obstructed with an opaque lens. To precisely control retinal light intensity, participant worn trial lens frames on which an artificial pinhole pupil of 3 mm was positioned at the slot closest to the eye and the other slots were used for refraction lens and neutral density filters. There were 5 filter conditions: 0 (no filter), 0.5, 1.0, 1.5 and 2.0 log units. Since the luminance intensity was 50 cd/m^2^ viewed through an artificial pupil of 3 mm, the retinal luminance intensity were 353, 112, 35.3, 11.2 and 3.5 td, respectively. All methods were performed in accordance with relevant guidelines and regulations. The experimental procedures were approved by the Comité de Protection des Personnes Ile de France V. Informed consent was obtained from both participants prior to the experiment.

### Apparatus

The stimuli were presented on a 22.5-inch LCD monitor designed for psychophysics (VIEWPixx) with a refresh rate of 120 Hz. At the viewing distance of 2 m, the spatial resolution of the display was 128 pixels/degree of visual angle. The monitor was the only light source in the room. The output intensity of each color gun was carefully linearized (gamma correction). The Noisy-bit method^9^ implemented independently to each gun made the 8-bit display perceptually equivalent to an analog display having a continuous luminance resolution.

### Stimuli and procedure

The stimuli and procedure were highly similar to the ones of our previous study on motion sensitivity^1^. The task consisted in discrimination the drifting direction (left or right) of a vertically oriented grating of 0.5 cycles per degree by pressing one of two keys. The signal was presented for 250 msec plus on and off half-cosine ramps of 125 msec each, for a total of 500 msec. The spatial window of the signal had a diameter of 4 degrees of visual angle plus a half-cosine of 1 degree of visual angle.

For each temporal frequency (0.9375, 1.875, 3.75, 7.5, 15 and 30 Hz), contrast threshold was measured with and without high noise. The noise used was truncated-filtered noise^10^ with an ideal low-pass cutoff at 2 cycles per degree and a truncation threshold at 1 standard deviation, refreshed at 120 Hz and had a maximal contrast of 0.5. This noise had a flat energy spectrum of 81 μ seconds degrees^2^ up to 60 Hz, which is greater than the critical frequency fusion so it was considered temporally white, and up to 2 cpd, which was 2 octaves above the signal frequency. When present, the noise was continuously displayed and covered the entire screen (i.e., spatiotemporally extended) to avoid triggering a processing strategy shift between no and high noise^11^,^12^.

Contrast thresholds were measured using a 3 down 1 up staircase procedure^13^ with step size of 0.1 log and were interrupted after 12 inversions. For each staircase, the threshold was estimated as the geometric mean of the last 8 inversions. Each of the 60 conditions (6 temporal frequencies X 2 noises X 5 luminance intensities) was performed 3 times and the threshold for each condition was estimated as the geometric mean of the 3 threshold estimates (i.e., 3 staircases).

Because observers had to be adapted to the luminance intensity, the order of the testing was blocked relative to the luminance intensity. Each block consisted of 12 staircases (6 temporal frequencies X 2 noises) performed in pseudo-random order and the 15 blocks (5 luminance intensities X 3 staircases) were also performed in a pseudo-random order. Adaptation time was set at least 20 minutes for the two lowest luminance intensities and at least 2 minutes for the three highest. A pilot experiment revealed that this adaptation duration was sufficient for performance to stabilize.

## Additional Information

**How to cite this article**: Allard, R. and Arleo, A. Reducing luminance intensity can improve motion perception in noise. *Sci. Rep.* **7**, 43140; doi: 10.1038/srep43140 (2017).

**Publisher's note:** Springer Nature remains neutral with regard to jurisdictional claims in published maps and institutional affiliations.
